# Aggregatibacter aphrophilus Brain Abscess and Ventriculitis in a Young Healthy Woman: A Case Report and Literature Review

**DOI:** 10.7759/cureus.105642

**Published:** 2026-03-22

**Authors:** Paola Kaira, Oleksandr Semeniuk, Ahmed Abdelmonem, Lei Lynn

**Affiliations:** 1 Internal Medicine, George Washington University, Washington, DC, USA; 2 Infectious Diseases, George Washington University, Washington, DC, USA

**Keywords:** 16s rdna sequencing, aggregatibacter aphrophilus, brain abscess, low pressure hydrocephalus, ventriculitis

## Abstract

*Aggregatibacter aphrophilus* is a rare cause of endocarditis and brain abscesses. Common potential risk factors include dental manipulation or disease, upper respiratory infections, proximity to animals, and heart disease. We present a case of a young woman without apparent predisposing factors who developed a deep-seated *A. aphrophilus *brain abscess complicated by ventriculitis and post-infectious low-pressure communicating hydrocephalus. Diagnosis was established by cerebrospinal fluid (CSF) 16S rDNA sequencing and a serum Karius® cell-free DNA test, suggesting hematogenous spread. Management required a seven-week course of ceftriaxone, adjunctive steroids for refractory inflammation, and neurosurgical interventions, including external ventricular drainage and ventriculoperitoneal shunt placement. This report underscores the diagnostic and therapeutic challenges of this condition and highlights the role of molecular diagnostics in culture-negative infections.

## Introduction

Brain abscesses are life-threatening infections most commonly caused by *Streptococcus viridans*, *Staphylococcus aureus,* or anaerobes. They disseminate to the central nervous system through either contiguous spread from upper respiratory or dental infections, or hematogenous spread from heart or lung infections. Lesions most commonly involve the frontal and temporal lobes [1]. Clinical presentation is variable, with headache, fever, and focal neurologic deficits occurring inconsistently, which may delay diagnosis. Management includes prompt initiation of broad-spectrum antibiotics and surgical drainage when indicated, with microbiologic data guiding targeted therapy.

*Aggregatibacter aphrophilus*, formerly *known as Haemophilus aphrophilus*, belongs to the HACEK group (*Haemophilus, Aggregatibacter, Cardiobacterium, Eikenella, Kingella*), a set of fastidious Gram-negative bacteria with a shared propensity to cause endocarditis. Although implicated in 1.2-3% of infective endocarditis cases, other infections remain rare [2,3]. In epidemiologic studies of brain abscesses, *A. aphrophilus* was isolated in 2-7% of cases of pyogenic intracranial abscesses using cultures and in nearly 10% of cases with the addition of molecular techniques [4]. While pediatric cases are more common, risk factors in adults include dental manipulation, heart disease, or pet exposure [5]. We describe a case of a deep-seated *A. aphrophilus* brain abscess in a previously healthy young woman without identifiable risk factors, complicated by ventriculitis and low-pressure communicating hydrocephalus.

## Case presentation

History of present illness

A 27-year-old woman presented with one week of worsening headache, neck stiffness, photophobia, phonophobia, nausea, and subjective fevers. She denied seizures, vision changes, or head trauma. Past medical and surgical history was unremarkable except for an uncomplicated vaginal delivery six months prior and Mirena® intrauterine device (IUD) placement three weeks prior. She had no history of immunosuppression or dental disease. Social history revealed no smoking, alcohol, or illicit drug use, no animal exposures, and no recent travel. In the ED, her vitals were within normal limits. Her physical examination revealed neck stiffness and a positive Kernig sign. The oral cavity appeared normal. Over the next few days, she became febrile with a maximum temperature of 102.9 F and developed recurrent episodes of transient left-sided weakness along with horizontal diplopia. During these episodes, her neurological examination showed four out of five strength in the left upper and lower extremities, with otherwise normal reflexes and sensation. Ophthalmological examination was consistent with bilateral CN IV palsy.

Investigations

On admission, the patient was hemodynamically stable with neutrophil-predominant leukocytosis and elevated inflammatory markers. CSF obtained from lumbar puncture was clear and slightly viscous, with analysis showing white blood cell count (WBC) 20,940 × 10⁹/L (94% neutrophils), red blood cell count (RBC) 2,000 × 10¹²/L, protein 541 mg/dL, and glucose <20 mg/dL. CT head without contrast was unremarkable. Initial blood and CSF bacterial and fungal cultures were negative. She underwent an extensive infectious workup, including a meningitis/encephalitis panel, syphilis, cytomegalovirus, Lyme disease, Epstein-Barr virus, *Histoplasma capsulatum*, and Coccidioides; all results were negative. Immunocompetence was also assessed with human immunodeficiency virus, hepatitis panel, *Mycobacterium tuberculosis*, complement levels, antinuclear antibodies, and CSF oligoclonal bands, all of which were unremarkable.

**Figure 1:**
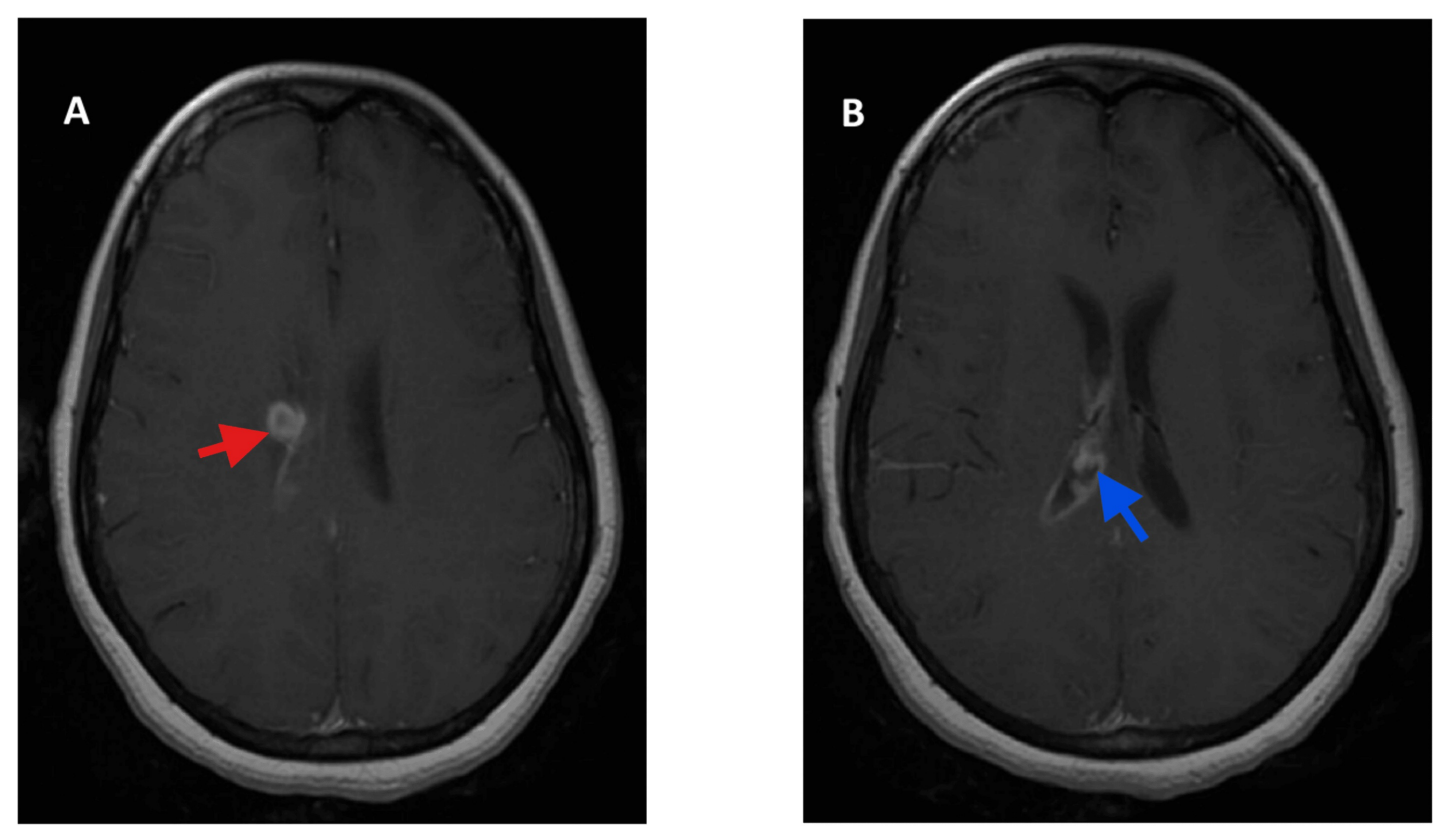
Initial MRI brain with and without contrast showing (A) 9 x 9 mm right centrum semiovale/caudate ring-enhancing lesion consistent with abscess (red arrow) and (B) debris in the right ventricle (blue arrow)

**Figure 2:**
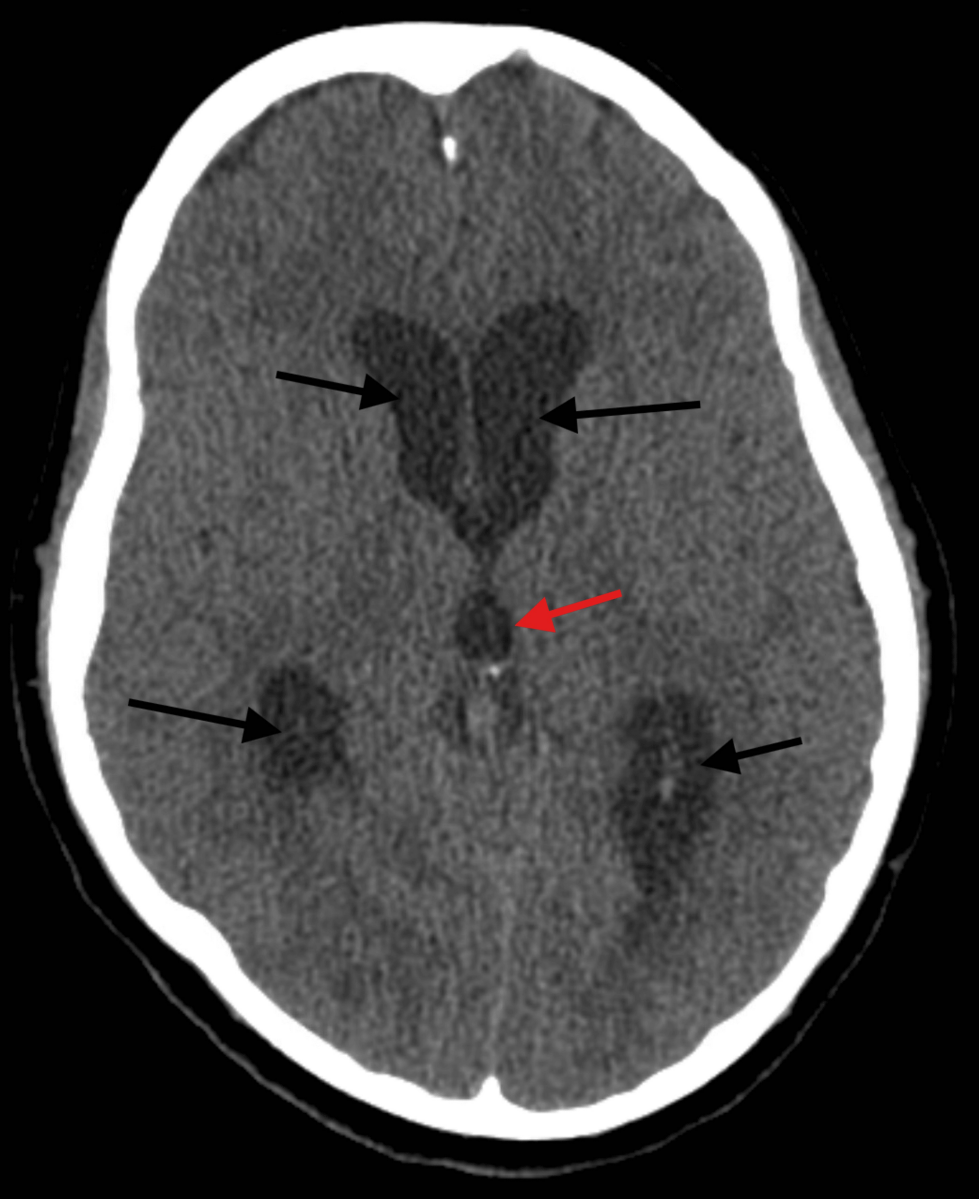
CT head without contrast showing communicating hydrocephalus with dilated lateral ventricles (black arrows) and third ventricle (red arrow)

A few days later, she developed new neurological symptoms, including episodic left-sided weakness and horizontal diplopia. Electroencephalography was negative. Repeat laboratory testing was suggestive of resolving infection (Table 1), again with negative cultures. Brain MRI identified a 9 × 9 mm right centrum semiovale/caudate ring-enhancing lesion consistent with an abscess, with ventriculitis and the presence of intraventricular debris, concerning for abscess rupture (Figure 1 A,B). Head and neck MRA showed diffuse arterial narrowing consistent with severe vasospasm. Advanced molecular testing with 16S rDNA polymerase chain reaction (PCR) of CSF and a serum Karius cell-free DNA assay identified *Aggregatibacter aphrophilus*. The patient subsequently developed worsening somnolence, and a non-contrast CT head showed communicating hydrocephalus (Figure 2), prompting emergent placement of a right external ventricular drain (EVD). Additional investigations, including echocardiography, CT angiography of the chest/abdomen/pelvis, and culture of a recently placed IUD, revealed no infectious source.

**Table 1:** Laboratory results

Laboratory Test	Initial Value	Repeat Value	Reference Range	Units (Unabbreviated)
White blood cell count (peripheral)	21 × 10⁹	—	4.0–11.0 × 10⁹	cells per liter
Neutrophils (peripheral)	91	—	40–70	percent
Lactic acid	1.5	—	0.5–2.2	millimoles per liter
Erythrocyte sedimentation rate	48	9.3	0–20 (female, varies by age)	millimeters per hour
C-reactive protein	10.7	5.1	<3.0	milligrams per liter
Cerebrospinal fluid white blood cell count	20,940 × 10⁹	7,337 × 10⁹	0–5 × 10⁹	cells per liter
Cerebrospinal fluid neutrophils	94	89	0	percent
Cerebrospinal fluid red blood cell count	2,000 × 10¹²	1 × 10¹²	0	cells per liter
Cerebrospinal fluid protein	541	331	15–45	milligrams per deciliter
Cerebrospinal fluid glucose	<20	<20	40–70	milligrams per deciliter

Treatment

On admission, empiric antibiotics were initiated with ceftriaxone 2 g every 12 hours, ampicillin 2 g every 4 hours, vancomycin 1.25 g daily, and dexamethasone 10 mg every 6 hours. This was narrowed to ceftriaxone and doxycycline after cultures returned negative, with discontinuation of doxycycline once Aggregatibacter aphrophilus was identified. Abscess drainage was not attempted due to its small size and deep location. The patient was started on dexamethasone 4 mg every 12 hours for suspected inflammation-mediated symptoms, resulting in marked improvement. After 10 days, she was switched to prednisone 40 mg twice a day, which was tapered slowly over the next month and a half. Due to the development of communicating hydrocephalus, a right EVD was placed and later converted to a left ventriculoperitoneal shunt after failed weaning attempts.

Outcome

**Figure 3:**
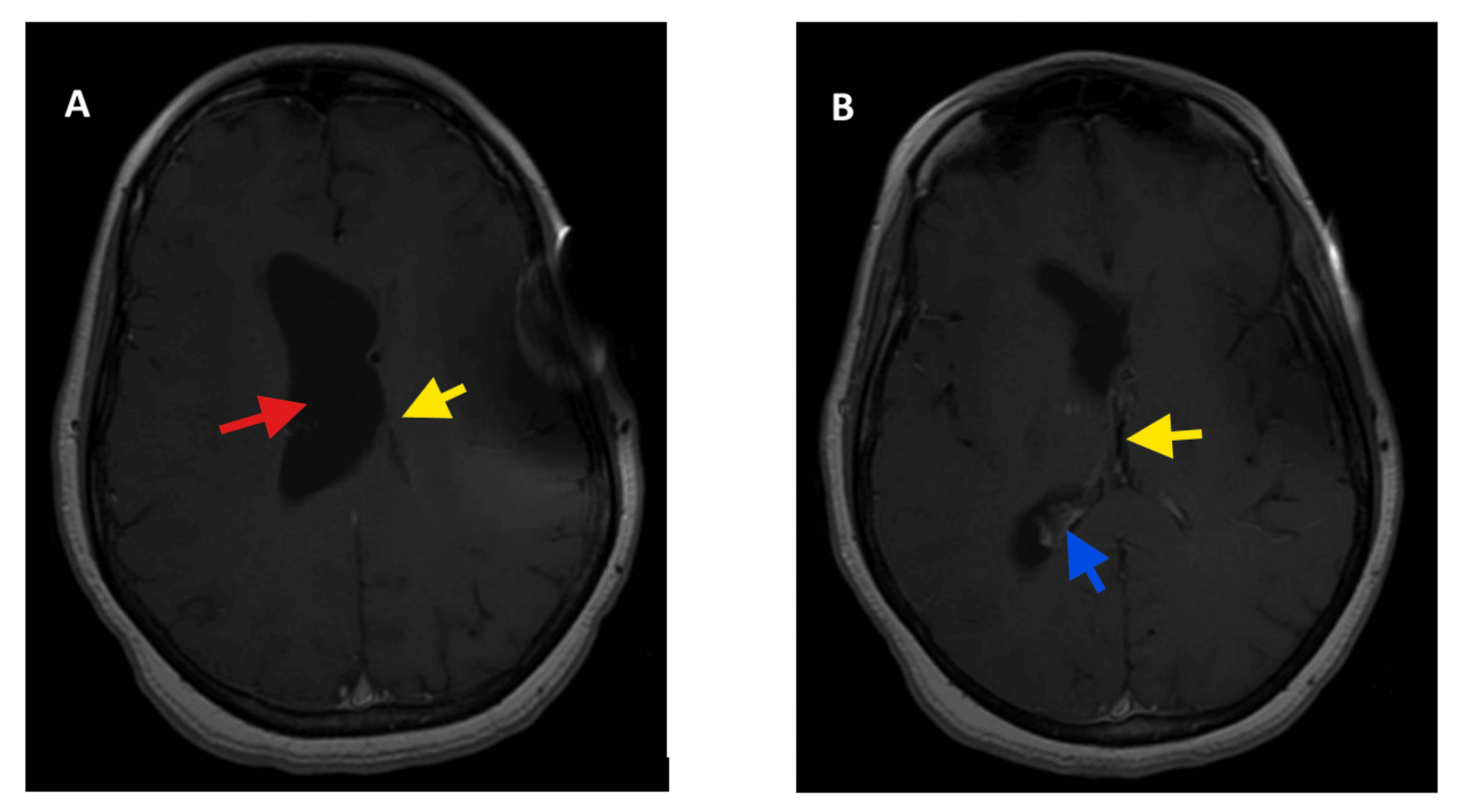
Repeat MRI brain with and without contrast showing (A) stable asymmetric ventriculomegaly involving the right lateral ventricle (red arrow) and (B) significantly decreased debris in the right ventricle (blue arrow). The yellow arrows highlight a decompressed left ventricle

Serial CSF studies showed steady improvement. The patient’s headaches, nausea, and transient weakness resolved after steroid initiation, with improvement in ventriculitis and cerebritis on repeat imaging. Resolving hydrocephalus was also noted following EVD placement. No identifiable source for the *A. aphrophilus* infection was found despite extensive workup. The patient completed a full seven-week course of ceftriaxone and was ultimately discharged in good clinical condition with a prednisone taper and a ventriculoperitoneal shunt. Two months after discharge, her diplopia had improved, and she otherwise reported only occasional headaches. A brain MRI with and without contrast showed stable appearance of asymmetric ventriculomegaly involving the right lateral ventricle and a significant interval decrease in ventricular debris (Figure 3 A,B). Due to her being on the lowest setting of her VP shunt valve and not having any clinical manifestation of overdrainage, the decision was made to continue monitoring.

## Discussion

We conducted a literature search on PubMed, Elsevier, and Google Scholar using various combinations of the terms “Aggregatibacter aphrophilus,” “Haemophilus aphrophilus,” “brain abscess,” “ventriculitis,” and “hydrocephalus.” The search yielded 12 readily accessible documented cases in the English literature, which were included in Table 2. The most reported risk factors included dental disease, oral manipulation, and animal exposure, particularly to dogs. Although the bacteria have been isolated from the mouths of patients’ pets in the past, no definitive zoonotic transmission has been established [6,7]. Notably, a quarter of reported cases involved young, otherwise healthy individuals without identifiable risk factors, similar to our patient. In our patient, *A. aphrophilus *was identified in both cerebrospinal fluid and blood using PCR-amplified 16S rDNA sequencing and serum cell-free DNA testing, respectively, suggesting a systemic infection with hematogenous seeding of the brain. Although recent childbirth and intrauterine device placement were considered potential sources, available evidence remains insufficient, and oral or respiratory origins remain the most plausible reservoirs.

**Table 2:** Review of Aggregatibacter aphrophilus brain abscesses in adult patients 5-ALA: 5-aminolevulenic acid, PCR: polymerase chain reaction, rDNA: ribosomal deoxyribonucleic acid, rRNA: ribosomal ribonucleic acid, mNSG: metagenomic next-generation sequencing, MALDI-TOF MS: matrix-assisted laser desorption ionization–time-of-flight mass spectrometry, IVDU: intravenous drug use, ICP: intracranial pressure.

Demographics	Co-infection	Risk Factor	Diagnosis	Abscess Size and Location	Medical Therapy	Complications	Reference, Year
54 M	None	Recent dental cleaning	PCR-amplified 16S rRNA sequencing	3 × 2.8 × 3.4 cm, right medial temporoparietal lobe	Ceftriaxone and metronidazole	Presented as glioblastoma multiforme with positive 5-aminolevulinic acid (5-ALA) fluorescence	[8], 2019
26 F	Eikenella corrodens	None	MALDI-TOF MS of abscess	Size unspecified, left frontal lobe	Ceftriaxone, metronidazole, and vancomycin	None	[9], 2023
Young male (age unspecified)	Eikenella corrodens	None	mNGS and MALDI-TOF MS of abscess	2.9 × 3.6 cm, right frontoparietal lobe	Ceftriaxone and meropenem	5h coma; Increased intracranial pressure requiring right frontotemporal parietal decompression of cranial bone flap	[5], 2024
43 M	None	IVDU, splenectomy	PCR-amplified 16S rDNA sequencing of abscess	Multiple scattered brain abscesses	Cefotaxime	Ventricular hemorrhage; ventriculitis; multiple lung abscesses; (no endocarditis)	[3], 2015
51 F	None	Atrial septal defect	Abscess culture	Size unspecified, right fronto-parietal occipital lobe	Meropenem followed by oral levofloxacin	The right lateral ventricle was narrowed significantly, 12 mm midline shift to the left. Required decompressive craniectomy due to cerebral herniation	[10], 2022
53 M	None	Recent history of infectious mononucleosis and streptococcus pharyngitis with full treatment	Blood cultures and abscess culture	3.8 × 3.7 x 3.4 cm, left frontal lobe	Ceftriaxone	Endocarditis	[11], 2019
42 M	None	Proximity to cats and poodle	Abscess culture	2.0 × 2.1 cm, left frontal lobe	Ceftriaxone and metronidazole	None	[12], 2017
73 M	None	Proximity to dogs and horses	PCR-amplified 16S rDNA sequencing of abscess	4 x 5.5 x 4.9 cm, right posterior parietal lobe	Ceftriaxone	Tonic-clonic seizures	[13], 2010
66 M	None	Recent dental abscess drainage	Abscess culture	Size unspecified, midbrain-thalamic region	Ceftriaxone and metronidazole	Worsening mentation leading to intubation and ventriculostomy due to increased ICP	[14], 2024
62 M	Actinomyces meyeri	Recent dental scaling	MALDI-TOF MS of abscess	4.7× 4.3 × 2.8 cm, left parietal lobe	Ceftriaxone and metronidazole	None	[15], 2021
58 M	None	Proximity to a poodle	Abscess culture	Size unspecified, right fronto-parietal lobe	Penicillin G	None	[6], 1986
28 M	None	None	Cerebrospinal fluid culture	10 mm × 9 mm, left midbrain tectum	Ceftriaxone	Initially misdiagnosed as a tectal glioma with obstructive hydrocephalus requiring EVD placement	[16], 2017

Anatomically, most *A. aphrophilus* brain abscesses occur in the frontal, parietal, or temporal lobes, with deep brain involvement being uncommon. Thalamic and basal ganglia abscesses are extremely rare, comprising only 1.3-6% of all brain abscesses [17]. Our patient’s abscess was located in the right centrum semiovale and caudate body, regions adjacent to the ventricular system and associated with an increased risk of ventriculitis and poor outcomes [17,18]. The left-sided weakness corresponded to involvement of the right corticospinal tracts, while the horizontal diplopia from CN IV palsy was likely due to inflammatory extension near the ventricles. This case highlights that even small abscesses can be high risk when located near critical structures, and that lesion size alone does not predict disease severity.

Management was challenging due to the deep, subcentimeter lesion and associated ventriculitis. Conservative medical therapy was favored over aspiration, given surgical inaccessibility and clinical stability. Ceftriaxone was used as definitive therapy based on its excellent CNS penetration and established efficacy against *A. aphrophilus* [9,19], consistent with the majority of reported cases. Adjunctive corticosteroids were initiated due to persistent ventriculitis on repeat brain imaging and inflammatory findings disproportionate to infectious markers. Although steroid use in brain abscess remains controversial, emerging evidence suggests no increase in mortality when adequate antimicrobial coverage is ensured, and in this case, steroids resulted in significant symptomatic improvement [19,20].

A notable complication in this patient was the development of post-infectious low-pressure communicating hydrocephalus, ultimately requiring ventriculoperitoneal shunt placement. Unlike the obstructive hydrocephalus more commonly associated with brain abscesses, this rare entity is characterized by ventriculomegaly without elevated intracranial pressure and improves only with negative-pressure drainage. Its pathophysiology is thought to involve inflammation-mediated changes to the subarachnoid space that lead to disruption of CSF flow and brain parenchymal viscoelasticity [21].

Finally, this case highlights the diagnostic value of hypothesis-independent molecular techniques in identifying fastidious organisms such as *A. aphrophilus*. While traditional cultures remain useful, molecular diagnostics, including 16S rDNA sequencing and cell-free DNA testing, were critical in establishing the diagnosis and confirming concurrent bacteremia, thereby supporting hematogenous spread. Early use of such tools may facilitate timely diagnosis and targeted therapy in similar cases.

## Conclusions

*Aggregatibacter aphrophilus* is a bacterium with low pathogenicity and is a well-established but rare cause of brain abscesses. Due to its fastidious nature, it may not grow in standard culture media, and hypothesis-independent molecular techniques are often required for prompt diagnosis, in addition to imaging modalities. Few cases of this condition have been documented in adults, and the clinical implications remain poorly understood. Therefore, close monitoring of each patient is imperative to mitigate potential complications, with a tailored approach to management. Future studies should also explore possible non-oral colonization sites for primary prevention.
